# Low Seroprevalence of SARS-CoV-2 in Rhode Island blood donors during may 2020 as determined using multiple serological assay formats

**DOI:** 10.1186/s12879-021-06438-4

**Published:** 2021-08-25

**Authors:** Daniel J. Nesbitt, Daniel P. Jin, Joseph W. Hogan, Jenny Yang, Haidee Chen, Philip A. Chan, Melissa J. Simon, Matthew Vargas, Ewa King, Richard C. Huard, Utpala Bandy, Christopher D. Hillyer, Larry L. Luchsinger

**Affiliations:** 1 New York Blood Center, Lindsley F. Kimball Research Institute, New York, NY USA; 2 Department of Biostatistics, Brown University, Providence, RI USA; 3 Rhode Island Department of Health, Providence, RI USA; 4 Rhode Island Commerce Corporation, Providence, RI USA; 5 Rhode Island State Health Laboratory, Providence, RI USA

**Keywords:** COVID-19, SARS-CoV-2, Seroprevalence, Rhode Island, Pandemic, Serology, Antibody

## Abstract

**Background:**

Epidemic projections and public health policies addressing Coronavirus disease (COVID)-19 have been implemented without data reporting on the seroconversion of the population since scalable antibody testing has only recently become available.

**Methods:**

We measured the percentage of severe acute respiratory syndrome- Coronavirus-2 (SARS-CoV-2) seropositive individuals from 2008 blood donors drawn in the state of Rhode Island (RI). We utilized multiple antibody testing platforms, including lateral flow immunoassays (LFAs), enzyme-linked immunosorbent assays (ELISAs) and high throughput serological assays (HTSAs). To estimate seroprevalence, we utilized the Bayesian statistical method to adjust for sensitivity and specificity of the commercial tests used.

**Results:**

We report than an estimated seropositive rate of RI blood donors of approximately 0.6% existed in April–May of 2020. Daily new case rates peaked in RI in late April 2020. We found HTSAs and LFAs were positively correlated with ELISA assays to detect antibodies specific to SARS-CoV-2 in blood donors.

**Conclusions:**

These data imply that seroconversion, and thus infection, is likely not widespread within this population. We conclude that IgG LFAs and HTSAs are suitable to conduct seroprevalence assays in random populations. More studies will be needed using validated serological tests to improve the precision and report the kinetic progression of seroprevalence estimates.

## Background

The Severe Acute Respiratory Syndrome Coronavirus (SARS-CoV)-2 pandemic is ongoing, with more than 30 million cases and over 550,000 deaths reported from Coronavirus disease (COVID)-19 in the United States as of early April, 2021 [1, 2]. Transmission models of SARS-CoV-2, based on numerous inferences of other immune responses to viral infections, suggest that infection may provide some immunity to reinfection [1, 3]. If true, the utility of serological tests to identify those who have acquired antibodies against SARS-CoV-2 (seroconversion) and the frequency of seroconversion in the population (seroprevalence) is a powerful tool with which to guide public health policies [4, 5]. It is critical to determine how many individuals have had COVID-19 and are thus likely to be immune, and differentiate them from those who have not been infected. These data are necessary to inform modeling projections and policy making that will allow an optimal approach to “reopening” a country, state, or region, and furthermore, these data must be accurate and reliable.

Serological assays rely on accurate recognition and ideally quantification of antibodies that recognize viral antigens specific to SARS-CoV-2. Optimal test characteristics include high levels of sensitivity and specificity. Coronaviruses have four major structural proteins; spike (S) protein (containing the S1 domain and RBD motif), nucleocapsid (N) protein, membrane (M) protein, and envelop (E) protein [6]. Research conducted on 2005 SARS-CoV-1 and Middle East respiratory syndrome Coronavirus (MERS-CoV), which are highly related to SARS-CoV-2, found that recovered individuals produced the strongest immunogenic antibodies against antigens of the S- and N-proteins [7]. Thus, the development of serological tests for SARS-CoV-2 antibodies has focused heavily on the detection of antibodies against these viral proteins. Antibody-based tests vary in both technology (platform) and target antigen (design). In May of 2020, the FDA announced a reversal in its emergency use authorization (EUA) and approval policies in order to help ensure that reliable tests are used to accurately measure seroconversion in a population. Some tests have received EUA but limited data is available. Considerable variability in test characteristics, particularly sensitivity, implies that there may not yet be an *ideal* test design and instrument platform. This also can lead to variability and potential bias in the estimation of the level of immunity in various locales or subpopulations [8, 9].

Multiple serological assays have been developed to detect SARS-CoV-2 antibodies from whole blood, plasma and serum. Essentially, three platforms of serological testing have been adopted: 1) enzyme linked immunosorbent assays (ELISA), 2) high-throughput serological assays (HTSA) and 3) lateral flow assays (LFA). ELISAs offer wide flexibility for research laboratories to select virtually any antigenic protein of interest and assay patient sera to provide highly sensitive, quantitative results. HTSAs are more suitable to clinical laboratories processing large volumes of samples. Although HTSAs offer a narrower selection of antigen choices, these platforms offer high-throughput capacity, high sensitivity and can be integrated into clinical lab testing facilities. LFAs also offer limited antigen diversity, but function with small volumes of whole blood, plasma or sera (1 drop, ~20uL) and require short test development times (≤30 min) allowing administration and test results at the point of care. As reagent supply, testing capacity and affordability vary across the country, the clinical community will undoubtedly resort to using multiple platforms to fill the demand.

Underreporting of COVID-19 cases may be occurring, which could inaccurately reflect the morbidity and mortality of SARS-CoV-2 [10]. The objective of this study was to assess the seroprevalence in a sample of blood donors in Rhode Island using commercially available serology tests [11]. To this end, consecutive blood donors were enrolled though the Rhode Island Blood Center (RIBC) into a pilot study with the goal of estimating seroprevalence for the population represented by those who donate blood on a regular basis. This pilot is part of a larger statewide effort to estimate seroprevalence, including a statewide community survey and testing on specific populations of interest.

## Methods

### Whole blood donors and sample preparation

From April 27, 2020 – May 11, 2020, consecutive Rhode Island Blood Center (RIBC) donors (*n* = 2008) received a 2-question survey and completed a blood or plasma donation. Donor blood samples were then tested using two commercially available serology tests and an in-house ELISA, described below. Plasma or serum was isolated from whole blood samples collected in silica clot activator tubes. Samples were extracted, aliquoted to minimize future freeze-thaw cycles, and stored at − 80 °C. Choropleth was generated in house from donor zip code prefix data using the web tool, http://www.heatmapper.ca/geocoordinate/ [12].

### Lateral flow ImmunoAssay (LFA)

LFAs were conducted using the Standard Q COVID-19 IgM/IgG Duo rapid immunochromatography test kit (SD Biosensor; South Korea) [13]. The kit contained two individual assay cartridges each with a detection band for IgG and IgM against SARS-CoV2 specific epitopes as well as an internal positive control. For each assay, 10 μL donor serum was applied to the sample pad, followed by two drops of proprietary running buffer according to the manufacturer’s instructions. After 15 min, a visual eye determination was made, and high-resolution images of the detection zone were taken and saved as. JPEG files. All tests were performed at room temperature.

### High-throughput serology assays

Serum samples were barcoded and dispatched to RIBC. Samples were analyzed using the VITROS Immunodiagnostic Products Anti-SARS-CoV-2 Total Ig Test with the VITROS 5600 (Ortho Clinical Diagnostics; USA). All assays were performed by trained RIBC employees according to the manufacturer’s standard procedures.

### In-house SARS-Cov2 binding-antibody ELISAs

Flat-well, nickel-coated 96 well ELISA plates (Thermo Scientific; USA) were coated with 2 μg/mL of recombinant S1 spike protein, nucleocapsid protein, or Receptor Binding Domain (RBD) spike protein specific to SARS-CoV-2 in resuspension buffer (1% Human Serum Albumin in 0.01% PBST) and incubated in a stationary humidified chamber overnight at 4 °C. On the day of the assay, plates were blocked for 30 min with ELISA blocking buffer (3% W/V non-fat milk in PBST). Standard curves for both S1 and RBD assays were generated by using mouse anti-SARS-CoV spike protein monoclonal antibody (clone [3A2], ABIN2452119, Antibodies-Online) as the standard. Anti-SARS-CoV-2 Nucleocapsid mouse monoclonal antibody (clone [7E1B], bsm-41,414 M, Bioss Antibodies) was used as a standard for nucleocapsid binding assays. Monoclonal antibody standard curves and serial dilutions of donor sera were prepared in assay buffer (1% non-fat milk in PBST) and added to blocked plates in technical duplicate for 1 h with orbital shaking at room temperature. Plates were then washed three times with PBST and incubated for 1 h with ELISA assay buffer containing Goat anti-Human IgA, IgG, IgM (Heavy & Light Chain) Antibody-HRP (Cat. No. ABIN100792, Antibodies-Online) and Goat anti-Mouse IgG2b (Heavy Chain) Antibody-HRP (Cat. No. ABIN376251, Antibodies-Online) at 1:30000 and 1:3000 dilutions, respectively. Plates were then washed three times, developed with Pierce TMB substrate for 5 min, and quenched with 3 M HCl. Absorbance readings were collected at 450 nm. Standard curves were constructed in Prism 8.4 (Graphpad Software Inc.) using a Sigmoidal 4PL Non-Linear Regression (curve fit) model.

### Estimated Seroprevalence statistical calculations

For each assay, seroprevalence was estimated using a Bayesian statistical method that adjusts for sensitivity and specificity of the specific test. The operating characteristics for the Ortho assay were obtained from the technical report distributed by the manufacturer; for SD Biosensor we relied on local validation data.

## Results

**Fig. 1:**
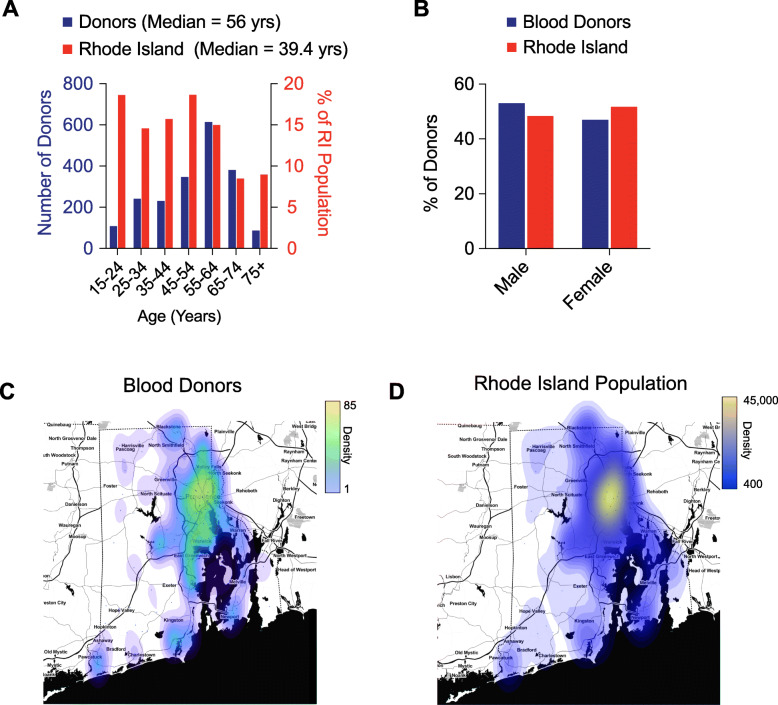
Demographics of Rhode Island Seroprevalence Donors. **A**; Distribution of seroprevalence donor age (blue bars) compared to RI population (red bars). *N* = 2008. **B**; Distribution of seroprevalence donor sex (blue bars) compared to RI population (red bars). N = 2008. **C**; Choropleth of zip codes for all seroprevalence blood donors. Choropleth was generated from donor zip code prefix data using the web tool, http://www.heatmapper.ca/geocoordinate/. **D**; Choropleth of zip codes for RI population (right). Choropleth was generated from donor zip code prefix data using the web tool, http://www.heatmapper.ca/geocoordinate/

A total of 2008 donor samples were collected for this study between April and May of 2020, just as the daily new case rates peaked in RI (https://ri-department-of-health-covid-19-data-rihealth.hub.arcgis.com/). We compared age, sex and race/ethnicity of the sample group to values reported for Rhode Island from the 2010 U.S. Census. The median age of donors was 56 years, significantly older than the Rhode Island median age of 39.4 years (Fig. 1A, Table 1). The sample had ~ 47% female donors compared to 52% statewide (Fig. 1B, Table 1). The distribution of donor Race/ethnicity was 84.7% white, 2.7% Hispanic/Latino and 0.50% Black/African American, compared to the state distribution of 81% white, 12.4% Hispanic/Latino and 5.7% Black/African American. A full comparison appears in Table 1 and Fig. 1. Notably, 9.3% of donors responded to ethnicity as ‘Declined’ or ‘Not Specified’. Finally, geographic location of donors associated with population density, such as Providence and Warwick, with lower representation in the western and coastal regions of Rhode Island (Fig. 1C, D). Thirteen donors were identified as convalescent plasma or whole blood donors that were aware of their seroconversion status prior to enrollment in the study and were removed from the analysis, which adjusted the total donors analyzed to 1996.

**Table 1:** Distribution of Study Donor Age, Sex and Ethnicity compared to 2010 Rhode Island Population

**Age Range**	**Study Donors**	**% Study Donors**	**RI Population**	**% RI Population**
15–24	108	5.38%	162,213	18.63%
25–34	241	12.00%	126,962	14.58%
35–44	230	11.45%	136,860	15.72%
45–54	347	17.28%	162,350	18.64%
55–64	614	30.58%	130,589	15.00%
65–74	381	18.97%	73,879	8.48%
75+	87	4.33%	78,002	8.96%
**Total**	2008		870,855	
Source: http://www.dlt.ri.gov/lmi/census/demo/agesex.htm				
**Gender**	**Study Donors**	**% Study Donors**	**RI Population**	**% RI Population**
Male	1064	53.01%	508,400	48.30%
Female	944	46.99%	544,167	51.69%
**Total**	2008		1,052,567	
Source: http://www.dlt.ri.gov/lmi/census/demo/agesex.htm				
**Ethnicity**	**Study Donors**	**% Study Donors**	**RI Population**	**% RI Population**
White	1700	84.66%	856,869	81.41%
Black or African American	10	0.50%	601,89	5.72%
American Indian & AK Native	5	0.25%	6058	0.58%
Asian	5	0.25%	30,457	2.89%
Hawaiian/Pacific Islander	0	0.00%	554	0.05%
Hispanic/Latino or Other Race, Alone	59	2.94%	63,653	6.05%
Two or More Races	20	1.00%	34,787	3.30%
Decline	23	1.154%	0	0.00%
Not Specified	186	9.26%	0	0.00%
**Total**	2008		870,855	
Source: http://www.dlt.ri.gov/lmi/census/demo/ethnic.htm				

**Fig. 2:**
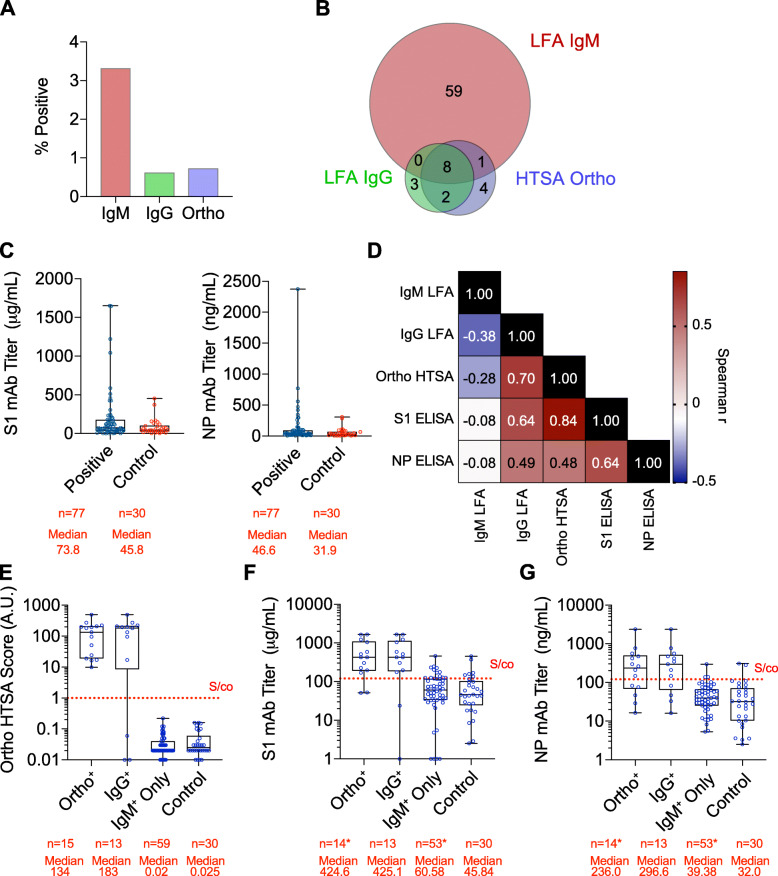
Serological Analysis of Rhode Island Seroprevalence Donors. **A**; Percent of donors testing positive using IgM LFA (red), IgG LFA (green) or Ortho HTSA (blue). **B**; Venn diagram of seropositive samples using IgM LFA, IgG LFA and Ortho HTSA. **C**; Monoclonal antibody quantification of all seropositive donors using S1 spike protein (left) and Nucleocapsid (N) protein (right) ELISA assays. Median values and number of samples are shown. **D**; Spearman correlation coefficients, r, between each serological assay. **E-G**; Evaluation of seropositive donors reactive to either the Ortho HTSA assay (Ortho^+^), the IgG LFA (IgG^+^) or only the IgM LFA (IgM^+^ only) and corresponding serological values using the Ortho HTSA platform (left) or the S1 ELISA (center) and NP ELISA (right) plaforms. Signal to cutoff (S/co) for each assay is indicated. Median values and number of samples are shown

To quantify seroprevalence in this sample, donor samples were tested with an HTSA platform (Ortho Clinical Diagnostics VITROS Total Ig Test) and an LFA platform (SD Biosensor IgM/IgG test). The IgM-only LFA assay yielded 68 positive tests for a 2.7% (95% CI 1.7 to 3.8%) seroconversion (Fig. 2A, Table 2). In contrast, the IgG-only LFA assay yielded 13 positive tests for 0.6% seroconversion (95% CI 0.3 to 1.1%) and was in agreement with the Ortho HTSA assay, which had 14 positives for a 0.6% seropositivity (95% CI 0.2 to 1.1) (Fig. 2A, Table 2).

**Table 2:** Antibody test results and seroprevalence estimates overall and by sex, age and race/ethnicity. Seroprevalence estimates reported in terms of posterior mode and 95% credible interval, calculated using Bayesian method that adjusts for test sensitivity and specificity. Estimates not reported for categories with 25 test results or fewer. Excludes 11 positive CP/WB Donors and 2 CP/WB donors that tested negative for all three tests. *Posterior mode calculated using a prior distribution having mode equal to the overall seroprevalence for IgM

		Number Positive			Seroprevalence Estimates (95% CI)		
	***N***	IgG	IgM	Ortho	IgG	IgM	Ortho
**Overall**	1996	13	68	14	0.6 (0.3 to 1.1)	2.7 (1.7 to 3.8)	0.6 (0.2 to 1.1)
**Sex**							
Men	1057	9	32	11	0.8 (0.4 to 1.6)	2.2 (1.1 to 3.8)	0.9 (0.3 to 1.8)
Women	939	4	36	3	0.3 (0.1 to 1.1)	3.1 (1.8 to 5.0)	0.1 (0 to 0.9)
**Age**							
15–34	348	2	4	5	0.6 (0.1 to 2.1)	0.2 (0 to 2.4)	1.3 (0.4 to 3.4)
35–64	1181	5	45	6	0.4 (0.1 to 1.0)	3.3 (1.9 to 4.8)	0.4 (0 to 1.0)
65+	467	6	19	3	1.2 (0.5 to 2.8)	3.4 (1.6 to 6.1)	0.5 (0.1 to 1.8)
**Race/Ethnicity**							
Asian	5	0	0	0			
Black/African American	11	0	0	0			
Hispanic/Latino	54	1	0	1	1.9 (0.4 to 10.2)	0.5 (0 to 7.7) *	1.8 (0.3 to 10.3)
Native American	5	0	0	0			
White	1688	10	57	11	0.5 (0.2 to 1.1)	2.7 (1.6 to 3.9)	0.5 (0.1 to 1.1)
Other / Multiple	24	0	2	0			
Unknown / Declined	209	2	8	2	0.9 (0.2 to 3.5)	3.0 (0.9 to 7.5)	0.8 (0.1 to 3.5)

In total, 3.9% of all samples (77 seropositive donors) were reactive for at least one test. To report overlap between test results, we constructed a Venn diagram (Fig. 2B, Table 2). Notably, ~ 76% of seropositive samples (59 of 77) were reactive only with the IgM-only LFA test. The remaining 0.9% of all samples (18 seropositive donors) showed a ~ 62% overlap between Ortho and IgG LFA assays (10 of 18 seropositive donors). Samples that showed at least 2 or more positive reactions was 0.55% (11 seropositive donors).

Donors completed a two-part questionnaire as to whether they had COVID-19 and the results of the diagnostic PCR test. However, error could be associated with this assessment due to inaccuracy of self-reported infection status as a result of false positive/negative test results or inaccurate donor reporting. Overall, 76 donors responded that they had received a diagnostic PCR test for COVID-19; of these, 13 donors tested positive while 63 tested negative (Table 3). Of those reporting positive PCR, 4/13 (44%) had positive IgM LFA, 9/13 (69%) had positive IgG, and 11/13 (85%) had positive Ortho test. Of those reporting negative PCR, 59/63 (94%) tested IgM negative, 61/63 (97%) tested IgG negative, and the same number (97%) tested Ortho negative. These limited data are in line with manufacturer-reported estimates of sensitivity and specificity. Importantly, the reliance on self-reported data must be interpreted with caution, and there was no ability to account for the time since infection, which could impact the sensitivity calculations.

**Table 3:** Serology Test Results stratified by reported PCR test result among SARS-CoV-2 Diagnostic PCR Test Respondents

		**PCR Result**		
		Positive	Negative	**Total**
**IgM**	Positive	4	4	8
	Negative	9	59	68
	**Total**	13	63	**76**
		**PCR Result**		
		Positive	Negative	**Total**
**IgG**	Positive	9	2	11
	Negative	4	61	65
	**Total**	13	63	**76**
		**PCR Result**		
		Positive	Negative	**Total**
**Ortho**	Positive	11	2	13
	Negative	2	61	63
	**Total**	13	63	**76**

The gold-standard in antibody quantification is the ELISA assay for its flexibility in antigen diversity and quantification methodology using monoclonal antibodies to generate standard curves. We designed in-house ELISA assays against S1 and NP specific to SARS-CoV-2 antibodies, since these antigens have been described to elicit the most immunogenic response to infection based on SARS-CoV and MERS research. We analyzed all 77 samples that were positive for any serological assay and 30 random samples that were negative for all serological assays as controls for S1 and NP antibodies. Surprisingly, S1 antibody quantification showed a median value of 73.8 μg/mL for seropositive samples compared to 45.8 μg/mL for seronegative controls (Fig. 2C) indicating moderate antibodies against S1 epitopes. Similarly, NP antibody quantification showed a median value of 46.6 ng/mL for seropositive samples compared to 31.9 ng/mL for seronegative controls, also indicating moderate antibodies against NP epitopes. However, there was ≥100-fold range of antibody values for seropositive samples in each ELISA test, suggesting that some of the seropositive samples, but not all, were significantly reactive in S1 and NP ELISA, which is highly predictive of neutralizing activity. Spearman’s correlation analysis of all five tests showed a high degree of positive association between ELISA, HTSAs and IgG LFA tests while IgM LFA test was negatively correlated (Fig. 2D). Thus, we hypothesized that samples reactive for either the IgG LFA and/or the Ortho HTSA may have higher ELISAs values than samples that were reactive only for IgM LFA test.

To investigate this, we evaluated seropositive donors that were reactive to either the Ortho HTSA assay (Ortho^+^), the IgG LFA (IgG^+^) or only the IgM LFA (IgM^+^ only). As expected, the median Ortho HTSA value was 10^4^ higher for both the Ortho^+^ and IgG^+^ groups compared to the IgM^+^-only group (158.5 and 114.8 AU vs 0.02 AU, respectively) (Fig. 2E). Similarly, both S1 and NP ELISAs showed significantly higher median antibody concentrations for the Ortho^+^ and IgG^+^ groups than for the IgM^+^-only group (S1; 467.2 μg/mL and 363.9 μg/mL versus 60.5 μg/mL and NP; 320.4 ng/mL and 204.8 ng/mL versus 39.4 ng/mL) (Fig. 2F, G). Importantly, these results conclude that IgG LFA and Ortho HTSA assays, but not the IgM LFA assay, correlate with immunogenic antibodies specific to SARS-CoV-2 as detected by ELISA.

## Discussion

This is among the first studies to evaluate statewide seroprevalence using blood donations. COVID-19 antibody testing has entered public discourse as an important metric in determining the population seroprevalence of SARS-CoV-2. Ultimately, the application of antibody testing could be clinically informative as to the degree of immunity afforded incurred by recovered patients or to that of future vaccinated individuals. However, we recognize the limitations of the current study include generalizability and limited demographic and other data of the blood donors that may be important. In fact, seroprevalence has been suggested to be higher in specific racial/ethnic communities based on recent studies [14]. Thus, more inclusive and complete seroprevalence studies will need to be performed in the future.

The application of antibody testing could be clinically informative as to the degree of antiviral activity incurred by recovered patients or to that of future vaccinated individuals. Seroprevalence studies have the ability to provide two important metrics: 1) the seroprevalence within a given population and 2) semi-quantification of specific antibodies to SARS-CoV-2 that may correlate with immunity. However, the latter estimation requires that an accurate methodology be adopted at the onset of the study. We recently completed a comprehensive analysis of SARS-CoV-2 serological test characteristics and comparison to antiviral neutralization activity using pseudoviral models [15]. In that investigation, HTSAs were shown to have superior performance characteristics and correlation with neutralizing activity compared to LFAs. It should be noted that the LFAs used in the prior study were different from the LFAs used in this study.

Among Rhode Island blood donors, we found the SD Biosensor IgG LFA and the Ortho HTSA assays both reported a ~ 0.6% estimated seroprevalence rate. Community seroprevalence estimates were only beginning to be made in May 2020 and questions surrounding testing platform sensitivity and specificity were still being evaluated thus providing no other data to compare our estimation. One study of randomly selected households across Rhode Island in May 2020 found an estimated seroprevalence of 2.1% (CI: 0.6–4.1%) [16]. To surveil the state, the authors performed a statewide cross-sectional household survey using HTSA or ELISA serological tests. The discrepancy in seroprevalence estimates between this study and ours can likely be attributed to the skewed donor demographics of blood donors, [17] where minorities are underrepresented. In another study, the first commercial laboratory seroprevalence estimate recorded by the CDC for the state of Rhode Island was 3.0% (CI: 1.2–5.5%) between July 30th and August 5th, 2020 [18]. Therefore, our lower seroprevalence estimate of ~ 0.6% during May 2020 would be consistent with the emergence of the second wave of infections which peaked on July 24th, 2020. However, seroprevalence calculations using a relatively limited number of donors coupled with low frequency of detection in the RI population during this period can introduce error into seroprevalence estimate calculations. Furthermore, our study is in agreement with a recent study showing relatively low seroprevalence in many metropolitan areas during this time period [19]. Overall, our study confirms a seroprevalence estimate consistent with other studies performed during this time period and show blood donation centers could be easily deployed to conduct rapid surveillance of regional populations to monitor future waves of COVID-19 and emerging pandemics.

It is tempting to speculate that low rates of seroprevalence is a logical result to the social distancing and mitigation policies that have been adopted by virtually the entire world. However, the SD Biosensor IgM LFA assay had very different performance characteristics, did not correlate with ELISA assays and reported a higher seroprevalence rate. The latter approximation would be similar to the Santa Clara seroprevalence rate reported in April of 2020, which found a seroprevalence rate of 2.5–4.2% using LFA assays [20]. However, since the IgM LFA assay correlated poorly with the Ortho HTSA assay, which we have previously shown to associate with neutralization activity and antiviral antibody effectiveness to prevent reinfection of cells with pseudovirus, [15] we conclude the SD Biosensor IgM LFA assay may not be informative as to an adaptive immune response to SARS-CoV-2. It should be noted that a concurrent SARS-CoV-2 serology study comparing the SD Biosensor LFAs to another LFA and a chemiluminescent assay concluded that the SD Biosensor IgM LFA had limited clinical utility, while the SD Biosensor IgG LFA performed very well across several distinct population sets and compared to the other assays [21]. However, since the infection status and time course of a potentially infected donor at the time of the blood donation was unknown, and IgM immunoglobin production precedes IgG antibody development, it is possible these donors were either recently infected or experienced a low exposure to SARS-CoV-2 but simply were not producing IgG at donation. Our results caution that seroprevalence rates could be miscalculated by as much as 5-fold depending whether an IgG or IgM LFA serology test is employed. Thus, prior assessment of serology assay performance should be considered before use in reporting rates of seroprevalence.

LFAs offer the convenience of rapid test results at the point of care and utilization of either whole blood, plasma or serum which makes deployment simple. In this study, we found that the SD Biosensor IgG LFA test provided reliable sensitivity to report seroprevalence. We found in this study that the SD Biosensor IgG LFA test also provided reliable sensitivity to report seroprevalence. However, LFAs do not yield semi-quantitative results which could be used to further understand the immunological range of responses within a study population. Therefore, HTSA platforms are better suited to quantify a wide range of antibody levels in a population while LFAs are suitable for low-cost, rural or studies designed for a limited interpretation of seroprevalence.

## Conclusion

In conclusion, we find the estimated seroprevalence of Rhode Island blood donors to be relatively low, approximately 0.6% during the month of May, 2020. Thus, we predict undiagnosed and asymptomatic infections are also likely to be low. Considering the possibility that this may be an underestimate of the statewide population, these conclusions draw important findings as it suggests that in the absence of a vaccine, “background” or “herd” immunity to also be low, now four months into the US pandemic, and thus the susceptible population remains at 95% or greater.

## Data Availability

All raw data presented in this manuscript is available on request from the corresponding author.

## Abbreviations

COVID-19: coronavirus disease of 2019
ELISA: enzyme linked immunosorbent assay
EUA: emergency use authorization
FDA: U.S. Food and Drug Administration
HTSA: high throughput serological assay
IgG: immunoglobin gamma
IgM: immunoglobin mu
LFA: lateral flow assay
NP: nucleocapsid protein
NYBC: New York Blood Center
PCR: polymerase chain reaction
S1: S1 domain of spike protein
SARS-CoV-2: severe acute respiratory syndrome
RBD: receptor binding domain of spike protein
RIBC: Rhode Island Blood Center
RIDOH: Rhode Island Department of Health
